# Supplementary material for: Interoperable slide microscopy viewer and annotation tool for imaging data science and computational pathology
Source: Nat Commun. 2023 Mar 22;14:1572. doi: 10.1038/s41467-023-37224-2 (PMC10033920; doi:10.1038/s41467-023-37224-2)
Supplement: Supplementary file 1 — Supplementary information [file 41467_2023_37224_MOESM1_ESM.pdf]

Interoperable slide microscopy viewer and annotation tool for imaging data science and computational pathology

February 13, 2023

Supplementary Figures

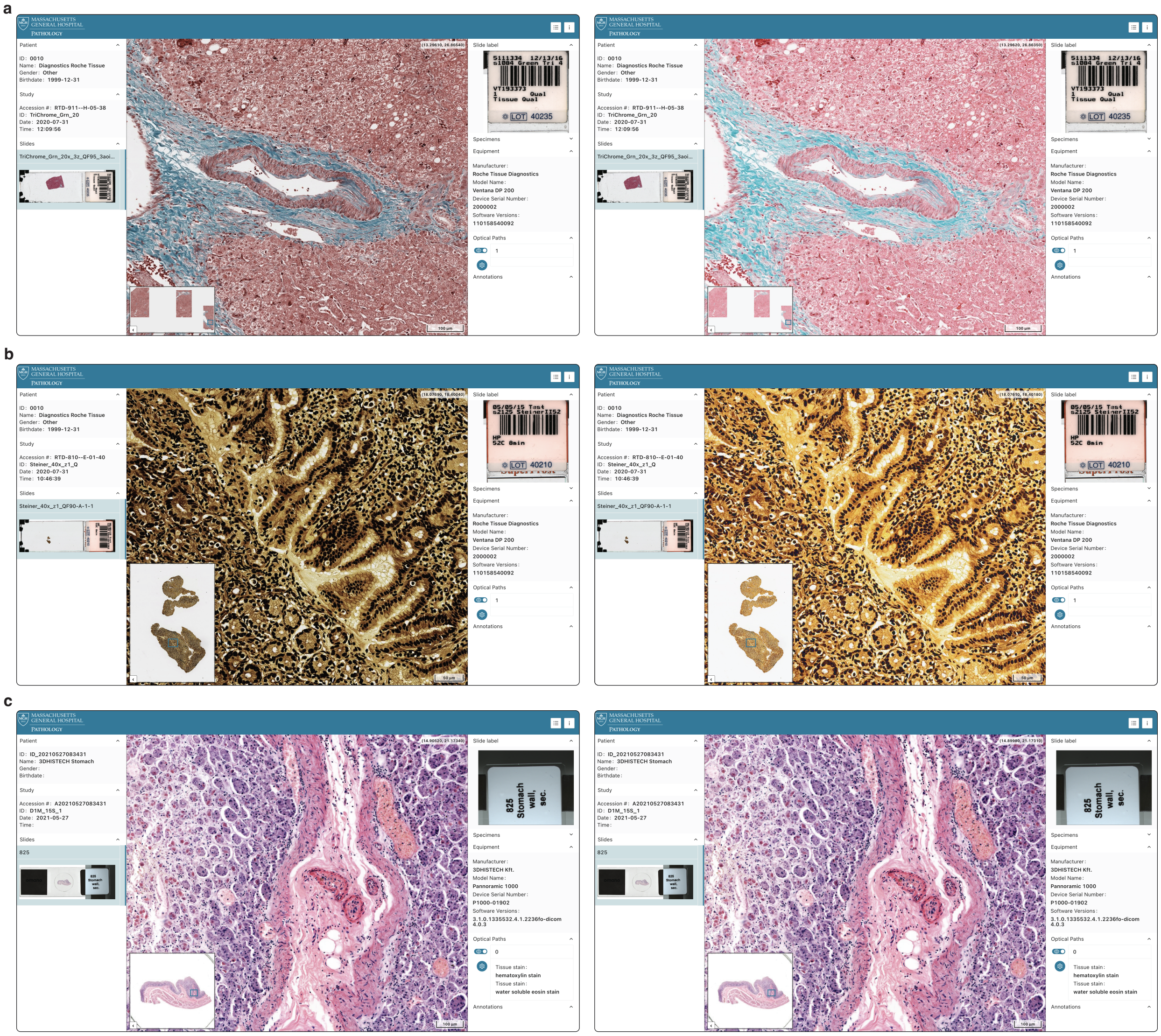

Supplementary Fig. 1. Correction of color images using ICC profiles. Shown are screenshots of Slim displaying color images displayed without (left) and with (right) color space transformations using device input ICC profiles included in DICOM image objects. **a**. Images of a trichrome stained tissue section specimen acquired by a Roche Tissue Diagnostics device. **b**. Images of a silver stained tissue section specimen acquired by a Roche Tissue Diagnostics device. **c**. Images of an H&E stained tissue section acquired by a 3DHISTECH device.

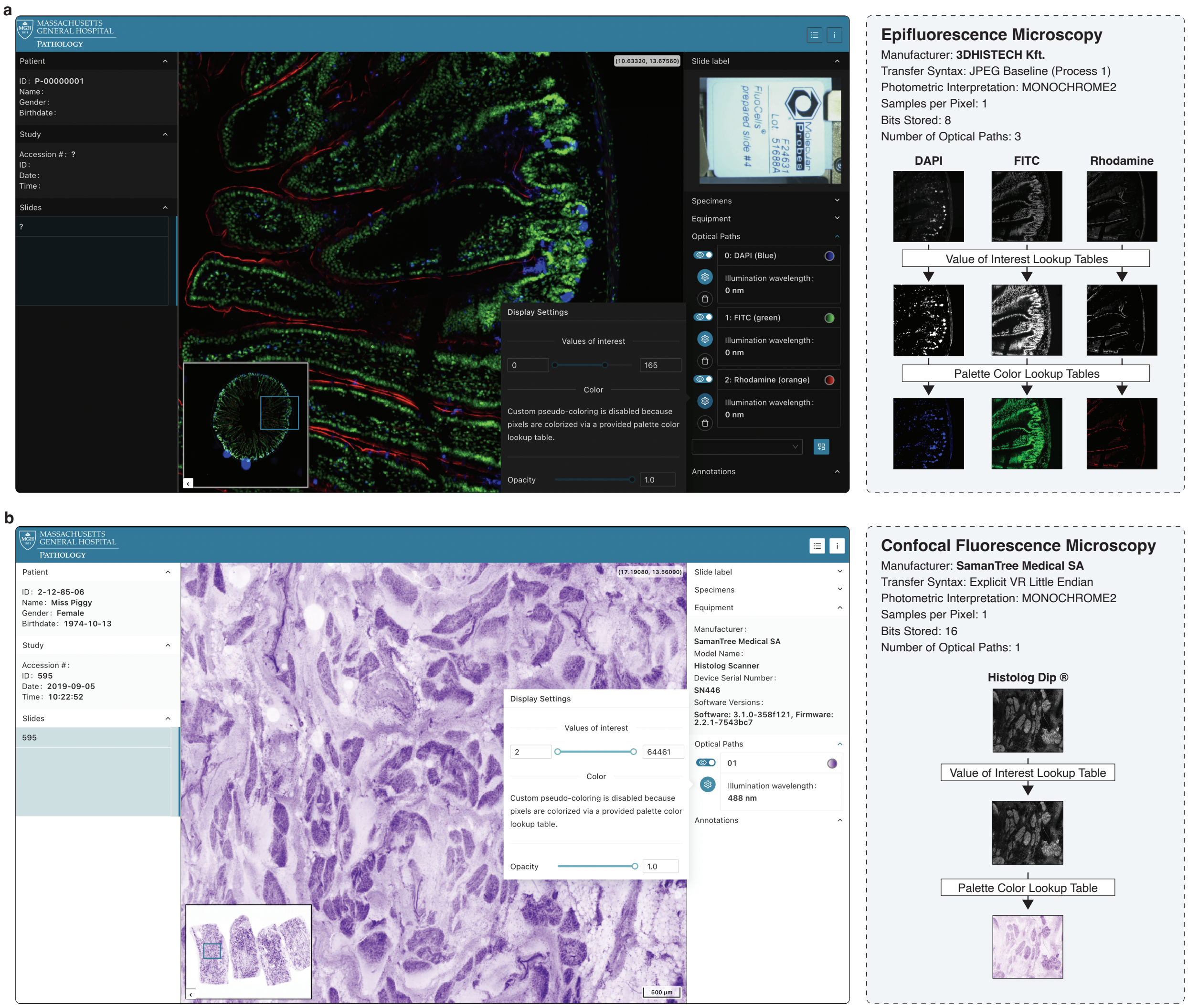

Supplementary Fig. 2. Pseudocoloring of grayscale images using palette color lookup tables. Shown are screenshots of Slim displaying grayscale images displayed after pseudocoloring using palette color lookup tables included in DICOM image objects. **a**. Multi-channel epifluorescence microscopy images of a DAPI, fluorescein isothiocyanate (FITC), and rhodamine stained tissue section specimen acquired by a 3DHISTECH Kft. device. Images of each channel are pseudocolored using the palette color lookup table for the corresponding optical path. **b**. Single-channel confocal fluorescence microscopy images of a Histolog Dip® stained intact tissue specimen acquired by a SamanTree Medical SA device. Images of the channel are pseudo-colored using the palette color lookup table for the optical path.
