# Supplementary material for: Interoperable slide microscopy viewer and annotation tool for imaging data science and computational pathology
Source: Nat Commun. 2023 Mar 22;14:1572. doi: 10.1038/s41467-023-37224-2 (PMC10033920; doi:10.1038/s41467-023-37224-2)
Supplement: Supplementary file 5 — Supplementary software [file 41467_2023_37224_MOESM5_ESM.zip › slim/public/404.html]

Slim
