# Supplementary figures and images for: Interoperable slide microscopy viewer and annotation tool for imaging data science and computational pathology
Source: Nat Commun. 2023 Mar 22;14:1572. doi: 10.1038/s41467-023-37224-2 (PMC10033920; doi:10.1038/s41467-023-37224-2)

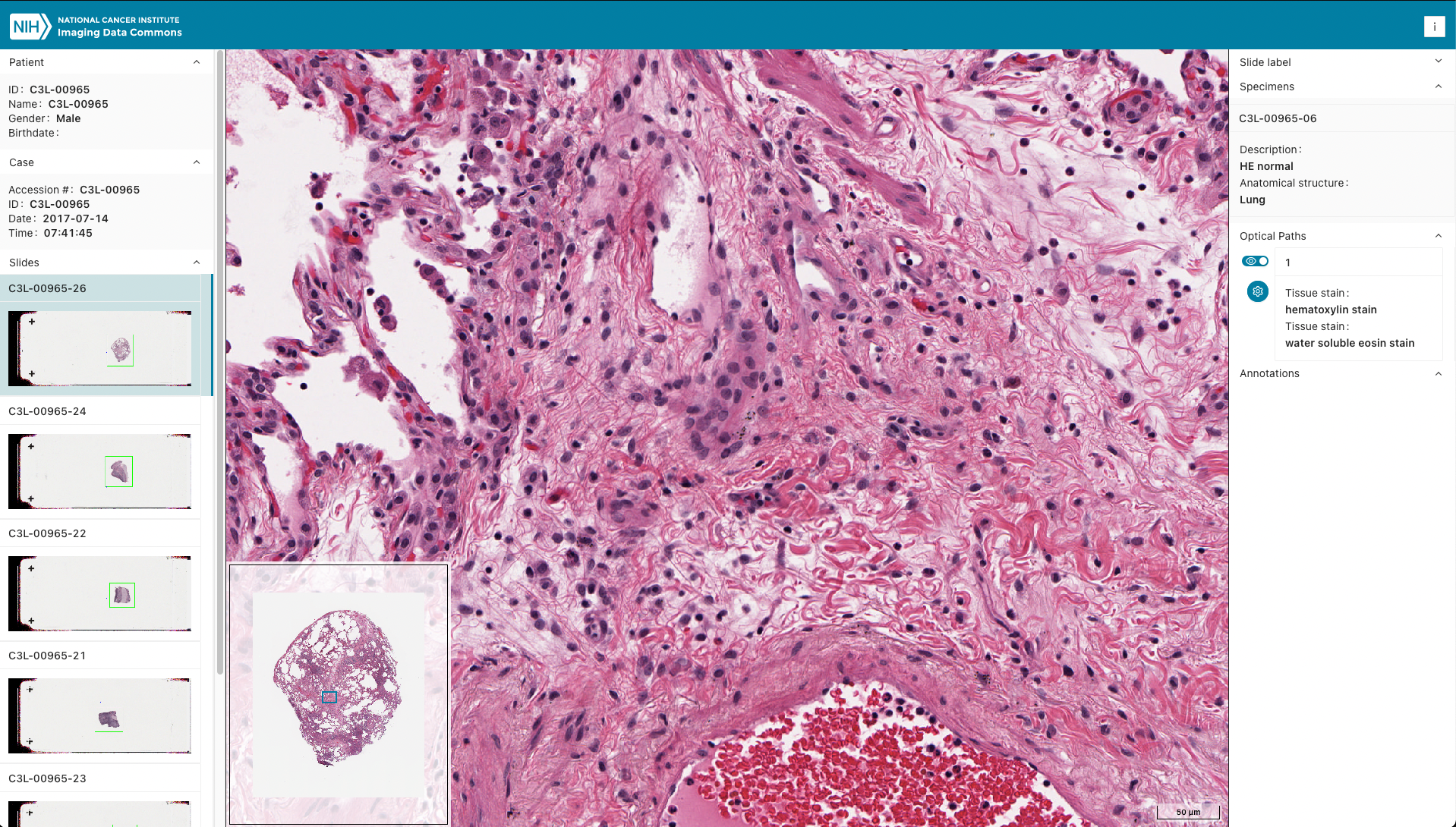

Supplement: Supplementary file 5 — Supplementary software [file 41467_2023_37224_MOESM5_ESM.zip › slim/docs/screenshots/IDC_CPTAC_C3L-00965-26.png]

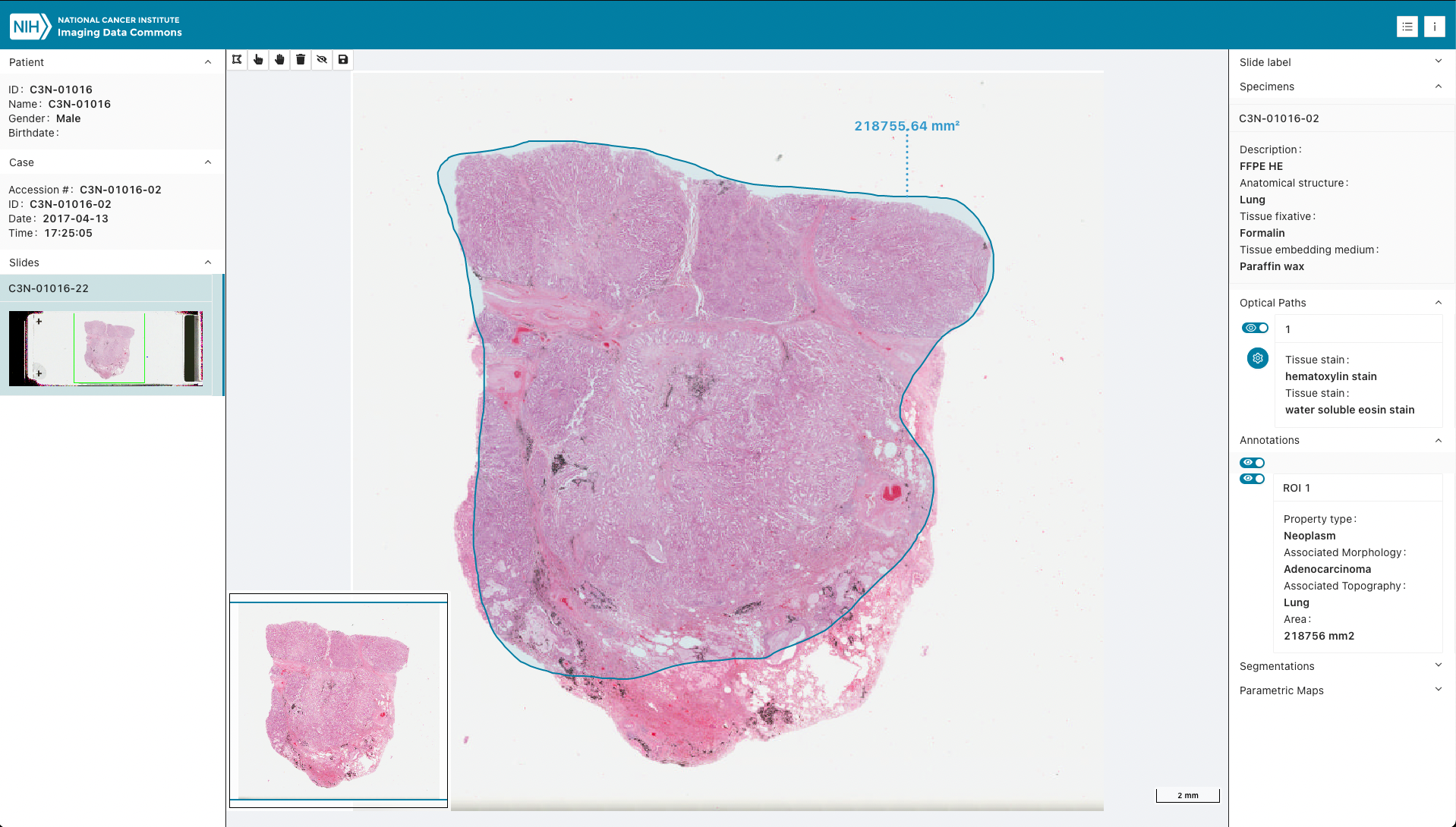

Supplement: Supplementary file 5 — Supplementary software [file 41467_2023_37224_MOESM5_ESM.zip › slim/docs/screenshots/IDC_CPTAC_C3N-01016-22_annotation.png]

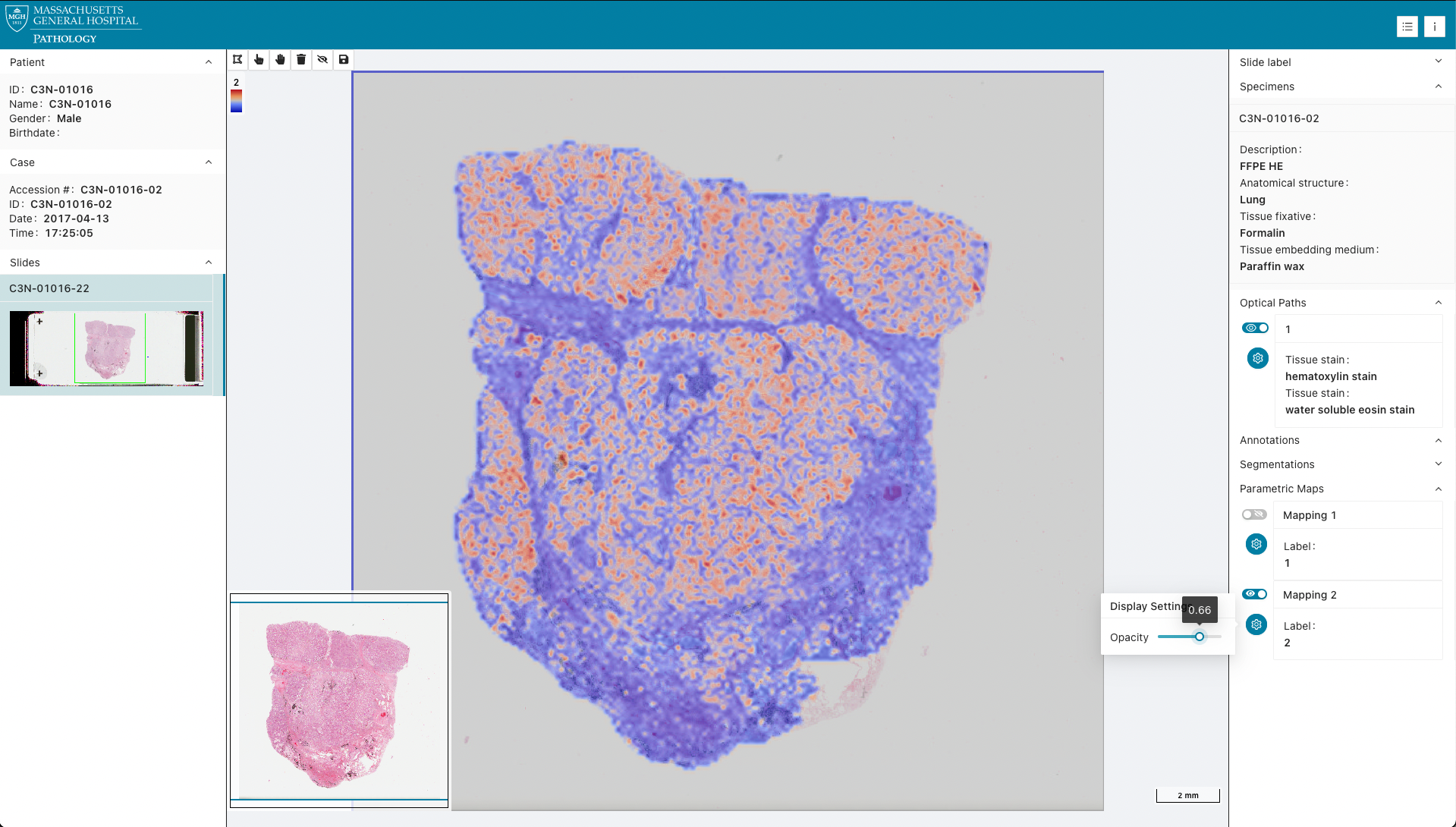

Supplement: Supplementary file 5 — Supplementary software [file 41467_2023_37224_MOESM5_ESM.zip › slim/docs/screenshots/IDC_CPTAC_C3N-01016-22_parametric_map.png]

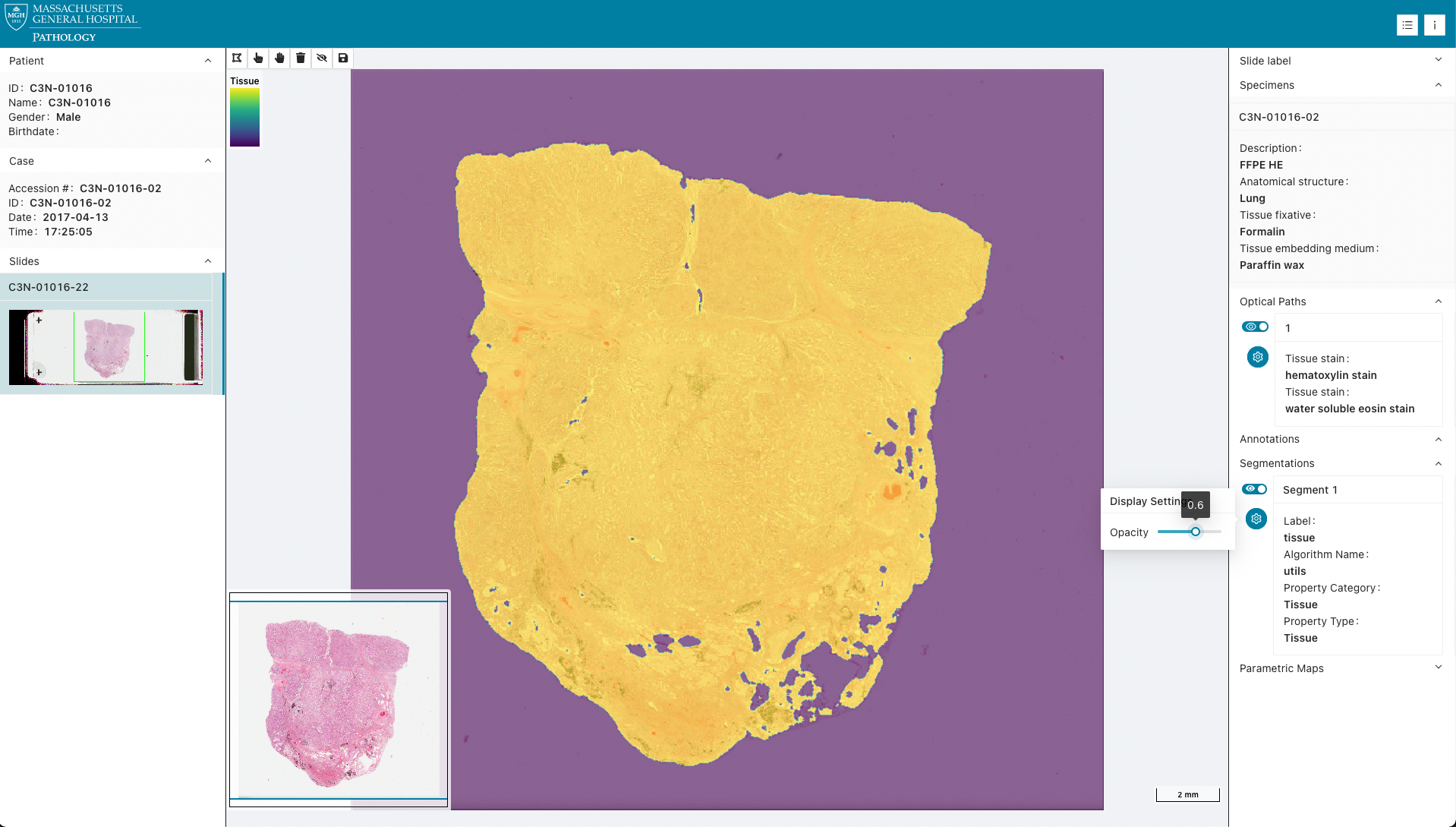

Supplement: Supplementary file 5 — Supplementary software [file 41467_2023_37224_MOESM5_ESM.zip › slim/docs/screenshots/IDC_CPTAC_C3N-01016-22_segmentation.png]

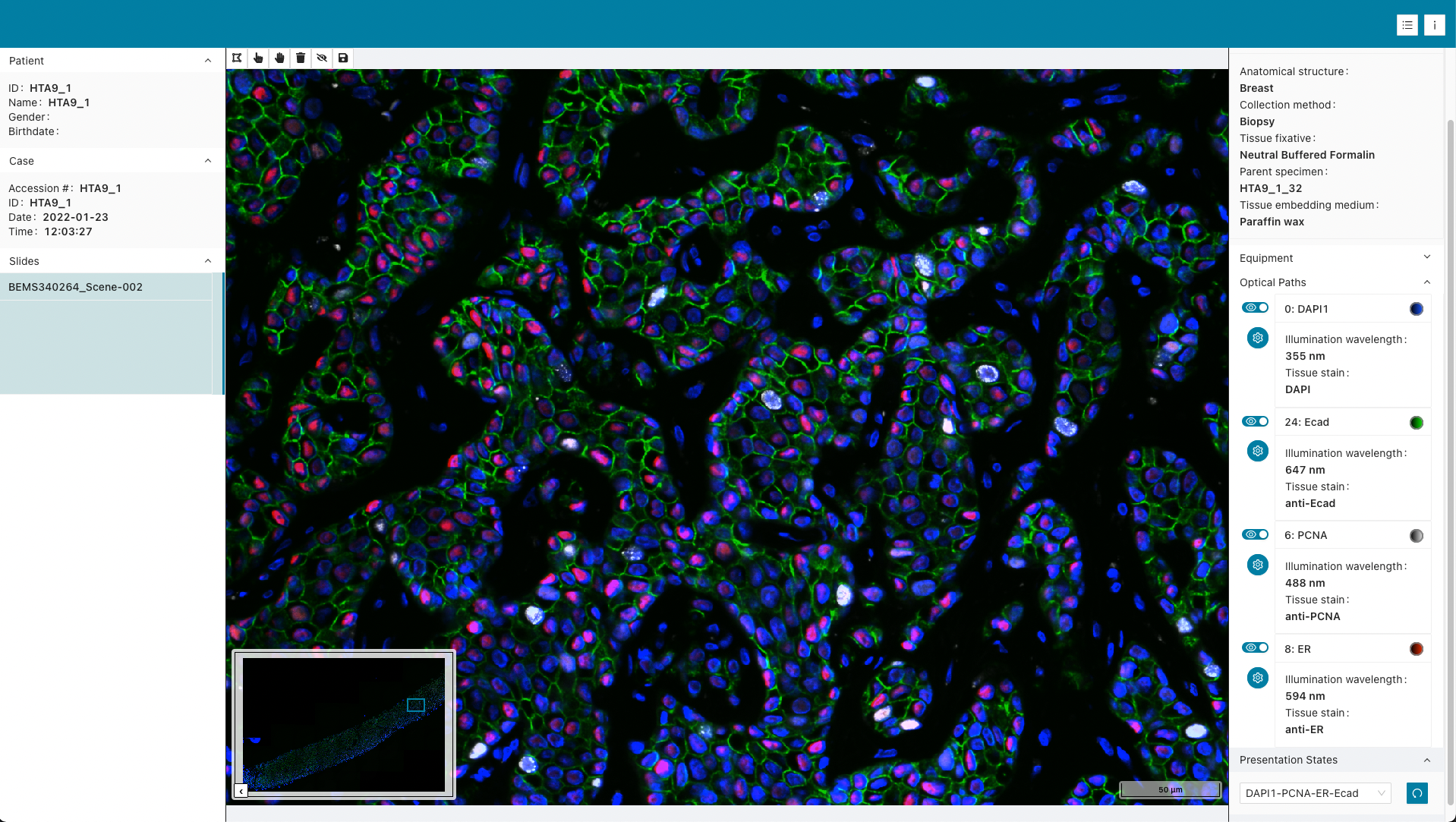

Supplement: Supplementary file 5 — Supplementary software [file 41467_2023_37224_MOESM5_ESM.zip › slim/docs/screenshots/IDC_HTAN_HTA9_1_32.png]

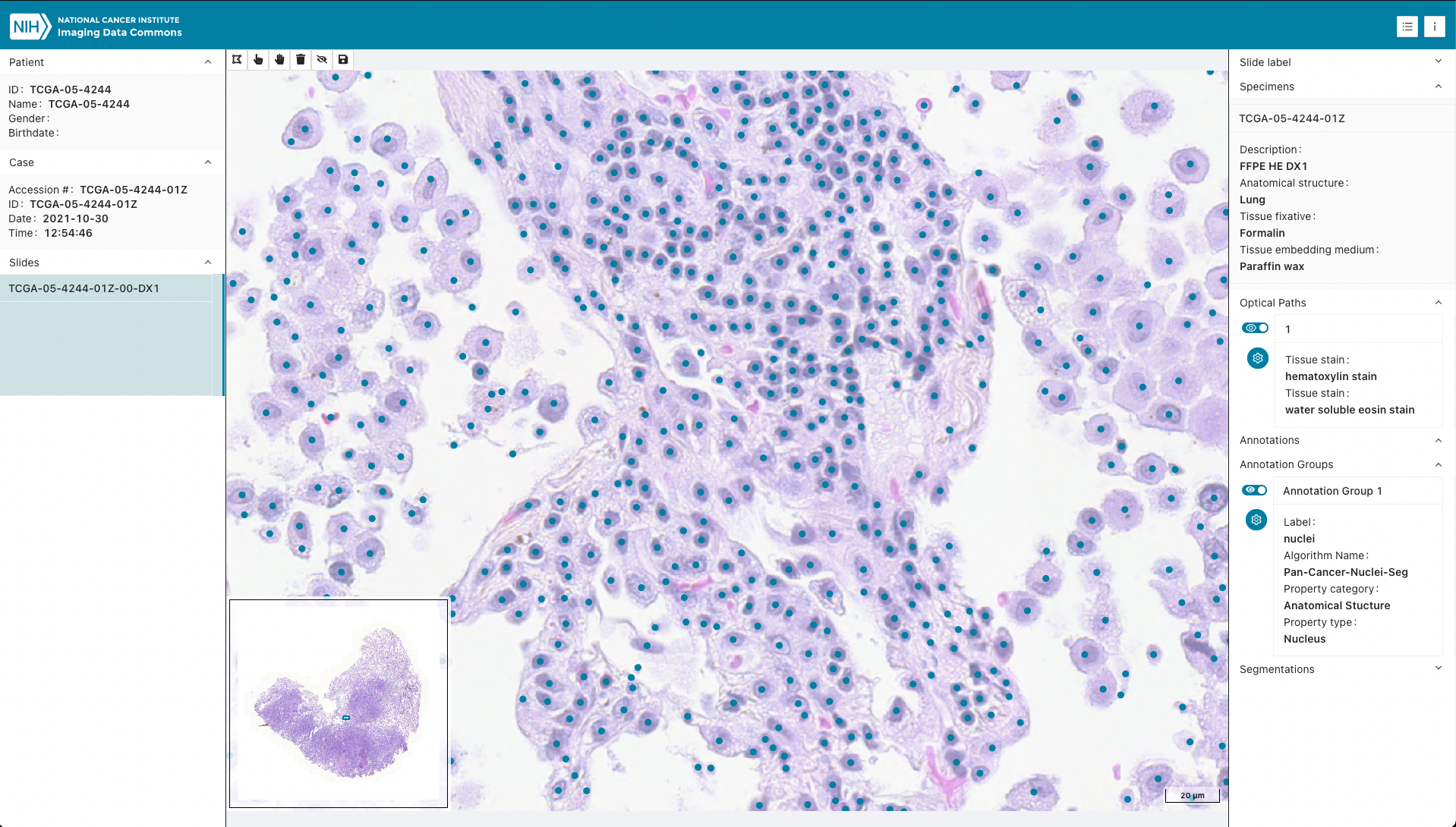

Supplement: Supplementary file 5 — Supplementary software [file 41467_2023_37224_MOESM5_ESM.zip › slim/docs/screenshots/IDC_TCGA_TCGA-05-4244-01Z-00-DX1_bulk_annotations.png]

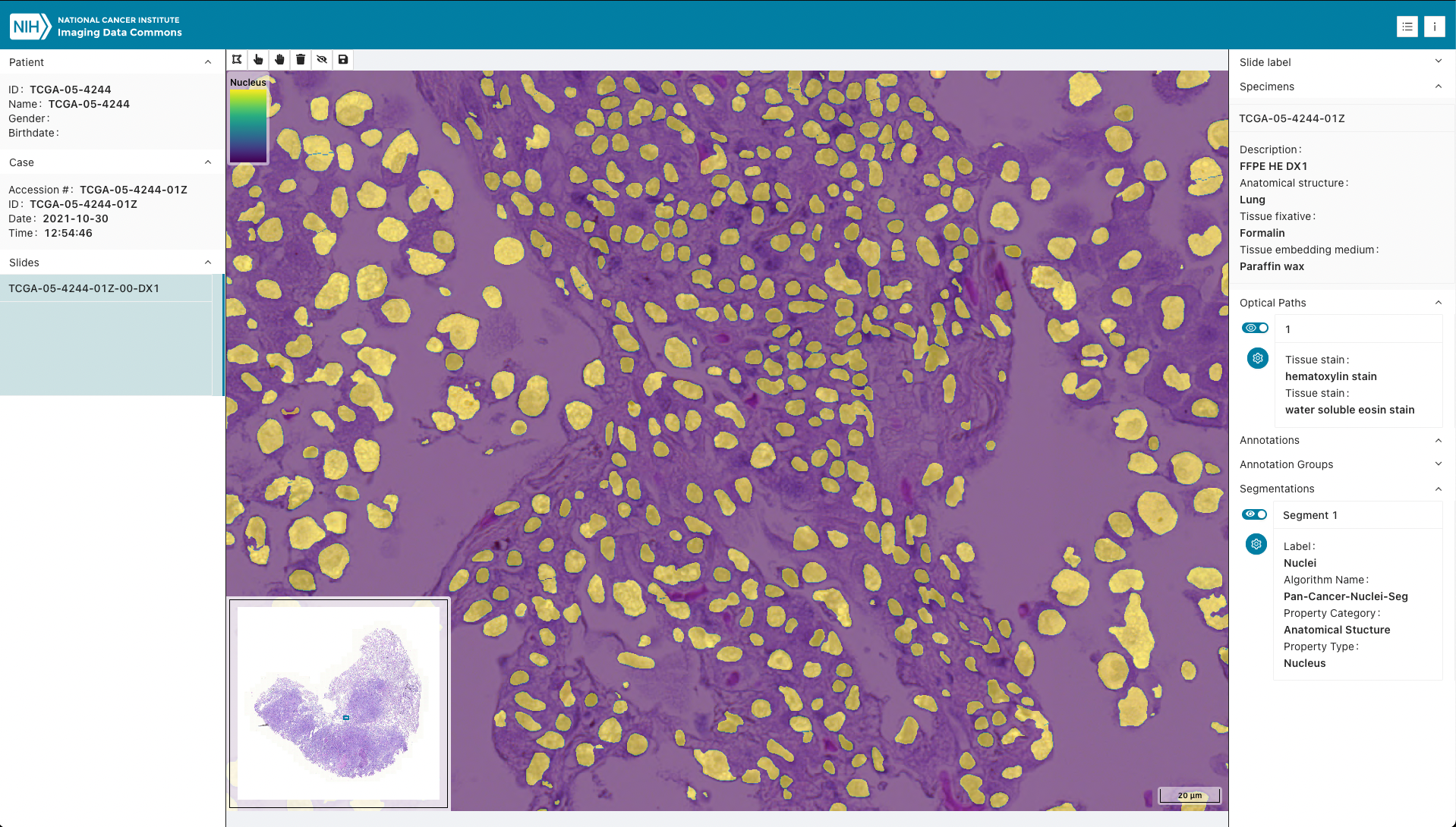

Supplement: Supplementary file 5 — Supplementary software [file 41467_2023_37224_MOESM5_ESM.zip › slim/docs/screenshots/IDC_TCGA_TCGA-05-4244-01Z-00-DX1_segmentation.png]

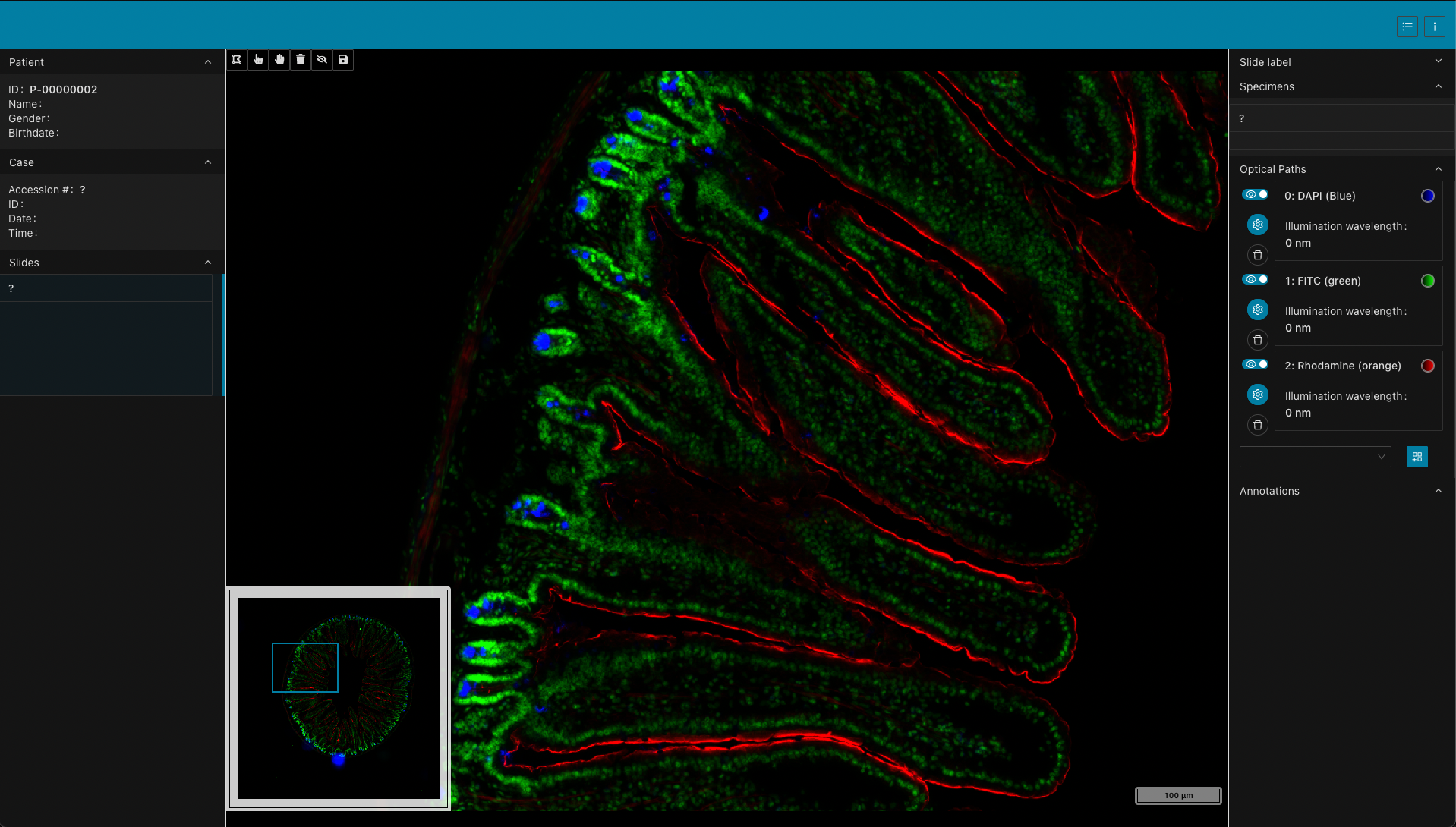

Supplement: Supplementary file 5 — Supplementary software [file 41467_2023_37224_MOESM5_ESM.zip › slim/docs/screenshots/NEMA_3DHISTECH_DAPI-FITC-Rhodamine.png]

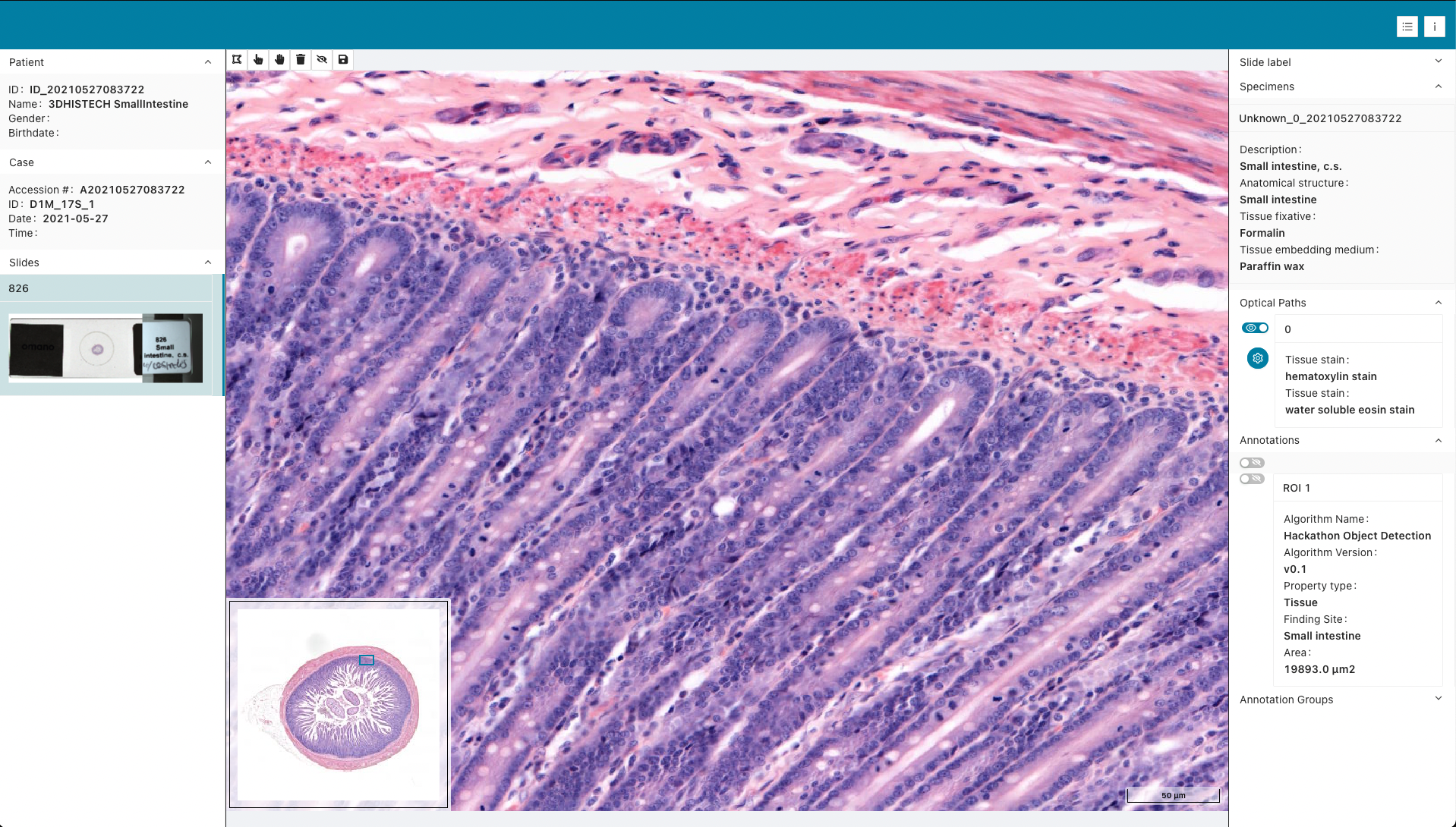

Supplement: Supplementary file 5 — Supplementary software [file 41467_2023_37224_MOESM5_ESM.zip › slim/docs/screenshots/NEMA_3DHISTECH_HE.png]

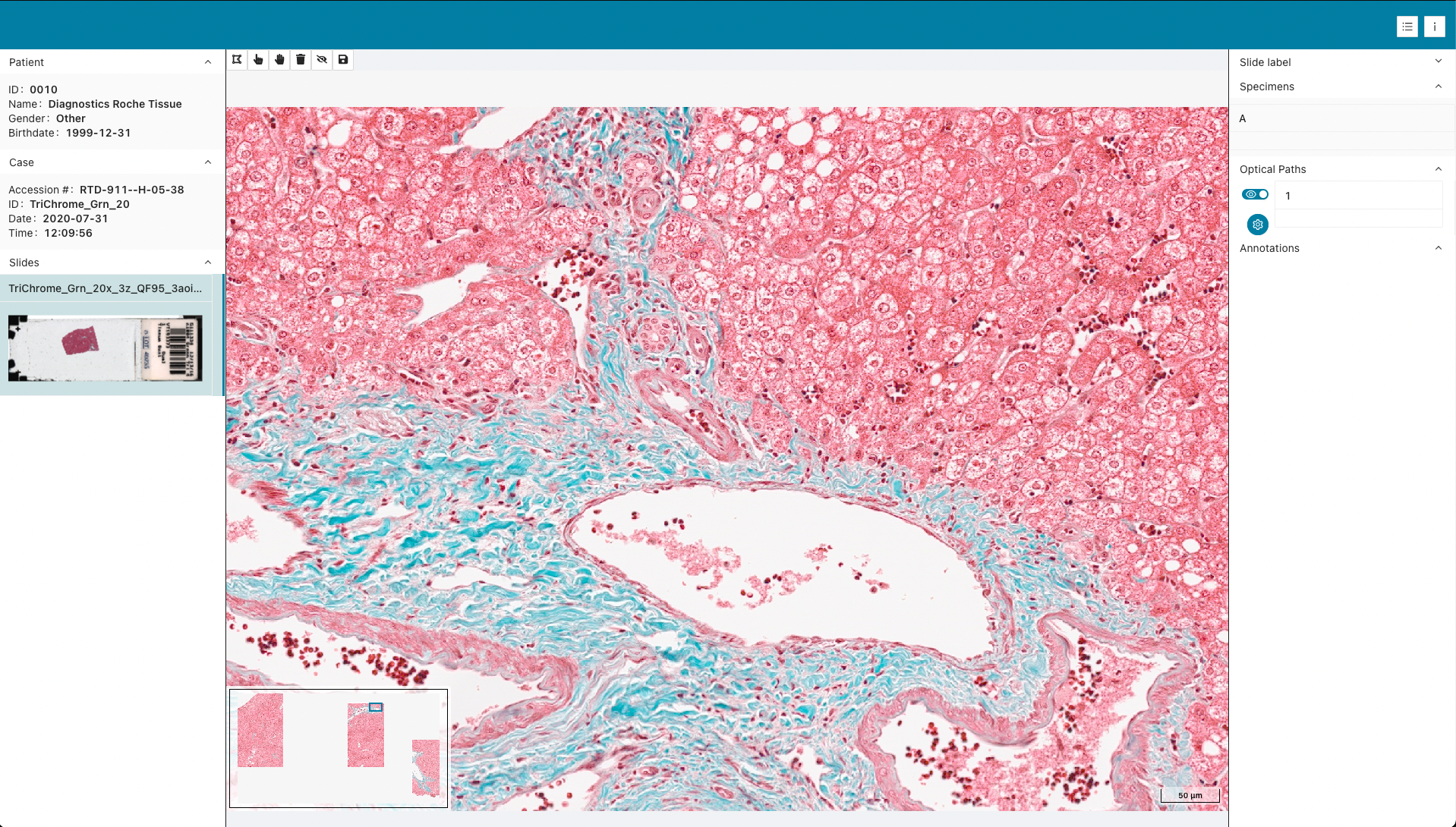

Supplement: Supplementary file 5 — Supplementary software [file 41467_2023_37224_MOESM5_ESM.zip › slim/docs/screenshots/NEMA_Roche_TriChrome.png]

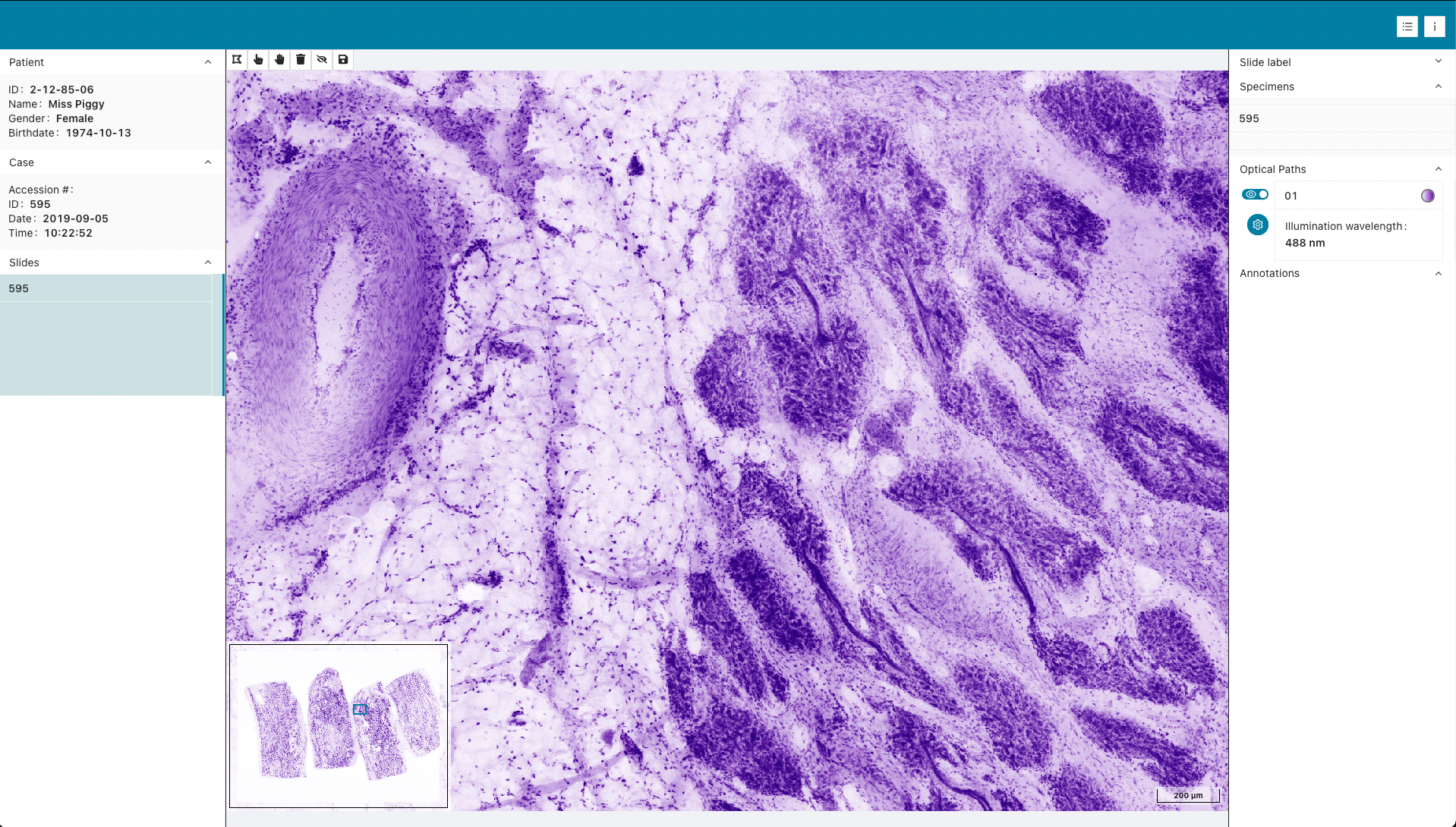

Supplement: Supplementary file 5 — Supplementary software [file 41467_2023_37224_MOESM5_ESM.zip › slim/docs/screenshots/NEMA_SamanTree_Histolog.png]

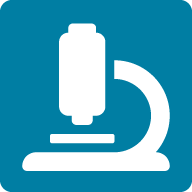

Supplement: Supplementary file 5 — Supplementary software [file 41467_2023_37224_MOESM5_ESM.zip › slim/public/logo192.png]

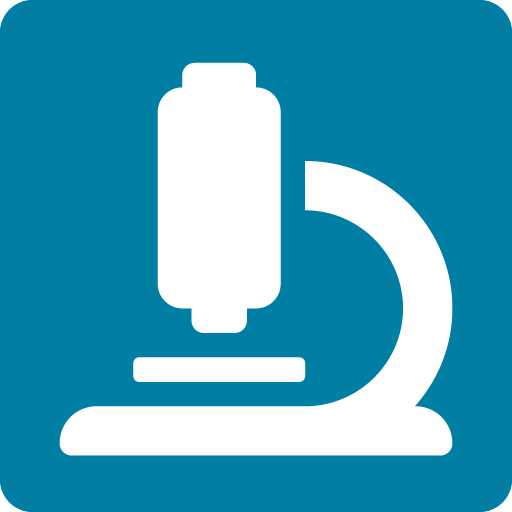

Supplement: Supplementary file 5 — Supplementary software [file 41467_2023_37224_MOESM5_ESM.zip › slim/public/logo512.png]
